# [18F] FDOPA standardized uptake values of brain tumors are not exclusively dependent on LAT1 expression

**DOI:** 10.1371/journal.pone.0184625

**Published:** 2017-09-22

**Authors:** Bérengère Dadone-Montaudié, Damien Ambrosetti, Maxime Dufour, Jacques Darcourt, Fabien Almairac, John Coyne, Thierry Virolle, Olivier Humbert, Fanny Burel-Vandenbos

**Affiliations:** 1 Department of Pathology, University Hospital, Nice, France; 2 UCA, Université Côte d’Azur, Nice-Sophia-Antipolis, France; 3 Department of Nuclear Medicine, Centre Antoine Lacassagne, Nice, France; 4 TIRO–UMR E 4320, University of Nice-Sophia-Antipolis, Nice, France; 5 Department of Neurosurgery, University Hospital, Nice, France; 6 UMR CNRS 7277-UMR INSERM 1091, Institute of Biology Valrose, University of Nice-Sophia-Antipolis, Nice, France; Kyung Hee University, REPUBLIC OF KOREA

## Abstract

[18F]-FDOPA is a labeled amino acid (AA) analog used for positron emission tomography (PET) which is gaining increasing interest in the evaluation of brain tumors (BT). The AA-transporter LAT1 has been shown to be involved in [18F]-FDOPA uptake. The aim of this study was to determine whether the [18F]-FDOPA uptake was correlated with level of LAT1 expression in BT. Twenty-eight BT (including 19 gliomas and 9 metastases) were investigated by [18F]-FDOPA-PET prior to surgery and by anti-LAT1 immunohistochemistry on surgical specimens. The quantitative [18F]-FDOPA measured parameters were SUVmax, SUVmean and SUVpeak. LAT1 expression was quantified using a score (0 to 400). A significant [18F]-FDOPA uptake was associated with a LAT1 score ≥ 100 (p = 0.02) but there was no linear correlation between intensity of [18F]-FDOPA uptake and score of LAT1 expression whatever the parameters considered. LAT1 expression alone is not sufficient to explain variation of intensity of [18F]-FDOPA uptake in BT.

## Introduction

Metastases and gliomas are the most common malignant tumours of the brain (BT). Oncological treatments are based on different modalities which may be used alone or in association: surgery when possible, radiotherapy and/or chemotherapy. Treatment modalities vary according to the group of tumors (metastases versus gliomas), the tumour location in the brain, the number of lesions, the stage of disease, the age and the status of the patient. Radiotherapy is usually advised in most BT, either focal in oligometastatic disease and in gliomas or as whole-brain irradiation in cancers with numerous brain metastases (BM). In both groups (BM and gliomas), the diagnosis of recurrence may be challenging with pseudoprogressive features on imaging due to radionecrosis. Due to the fact that some recurrent tumours may be re-treated by radiotherapy, chemotherapy or surgery, it is crucial to distinguish recurrence from radionecrosis.

MRI is the gold standard in the evaluation of brain lesions. However it is frequently insufficient to differentiate conclusively between tumour recurrence and radionecrosis. To overcome this problem the use of other techniques providing metabolic data is required. 18F-fluoro-deoxyglucose ([18F]-FDG) has been used very early in the history of positron emission tomography (PET) for the management of brain tumours. [1]. Differentiating increased tumour uptake from normal tissue is often difficult due to the physiological metabolism in the normal cortex. Labelled amino-acids (AA) and their analogues are particularly useful for imaging brain tumours because of their high accumulation in tumour tissues and their low uptake in the normal brain tissue [2]. Exact mechanisms of uptake of AA in brain tumours are currently imperfectly understood. It is generally accepted that the over-expression of the L-type amino-acid transporter 1 (LAT1) in tumour cells and their vasculature plays a major role [3–5]. LAT1’s transport capacity is independent of sodium and is variable according to extracellular pH, with activities increasing in low pH conditions [6,7]. In order to perform its role as an AA transporter, LAT1 needs the cofactor 4F2hc (or CD98), with which LAT1 forms a heterodimeric complex via a disulfide bond, although it has been shown that exclusive expression of LAT1 is sufficient to increase transportation activity, particularly in epithelial cells [8]. Since LAT1 is normally expressed at the blood brain barrier, AA tracers do not need blood brain barrier breakdown, unlike contrast media, in order to be taken up by brain lesions. The first radiolabeled amino acid used was 11C-methionine ([11C]-MET) [9]. Subsequently, AA labelled with 18F-fluorine were developed. Two of these have been developed and clinically tested so far: 18F-Fluoroethyl-L-Thyrosine ([18F]-FET) and 18F-Fluoro-L-DOPA ([18F]-FDOPA). They are both transported by LAT1 like [11C]-MET, but unlike [11C]-MET, they are not further metabolized. [18F]-FET and [18F]-FDOPA have shown very similar behaviour and usefulness in brain tumour imaging like [11C]-MET [10,11]. [18F]-FET and [18F]-FDOPA play an important role in current practice for patient management, bringing additional information to multimodality MR imaging especially for the early differential diagnosis between recurrence and post-treatment changes during follow-up [2,12,13].

In a number of studies, AA radiopharmaceuticals standardized uptake value (SUV) have been shown to be correlated with LAT1 expression in gliomas [14,15] and in primary central nervous system lymphoma [16]. Most studies assessing the correlation between AA uptake and LAT1 expression were performed with [11C]-MET [14,16]. In practice, [18F] FDOPA uptake intensity is variable from one BT to another and the mechanisms underlying uptake levels are not known. Only one study [15] found a correlation between SUVmean and LAT1 expression in a series of 6 gliomas. Recently, we demonstrated that LAT1 was overexpressed in almost all brain metastasis (BM) and LAT1 overexpression was specific for BM as compared to healthy brain and could explain the specific AA uptake by BM on PET-AA usually observed in clinical practice [17]. A recent study analyzed the role of LAT 1 in boronophenylalanine uptake and showed the selectivity of the radiopharmaceutical for LAT1 versus LAT2 and the balance between influx and efflux through LAT1 [18]. There is a need for more correlative analyses between *in vivo* [18F] FDOPA PET imaging and histopathology to improve understanding of [18F] FDOPA uptake mechanisms in order to refine its clinical use in particular in the difficult context of post treatment evaluation [2]. The aim of this present study was to analyze the correlations between [18F] FDOPA uptake intensity and the level of LAT1 expression in a series of 28 BT.

## Materials and methods

### Tissue and lesions

All the patients with a BT who underwent surgery less than 2 months after a [18F] FDOPA-PET from July 2013 to march 2015 were included in the present study. This study is an ancillary study of the prospective clinical trial IMOTEP (Registre des Essais Cliniques en cancérologie en France, Number RECF2013). This trial was approved by the ethical committee of the Centre Antoine Lacassagne on September 13, 2013. Informed consent was obtained from all individual participants included in the study. All patients gave written consent for the use of tumour sample in research. A total of 28 BT from 28 patients fulfilled inclusion criteria. The patient population comprised 15 men and 13 women with a mean age of 56 years (range 33–71). Twenty two patients had been treated initially with radiotherapy before surgery for suspected recurrence. The BT included 19 gliomas (13 glioblastomas, 2 grade II oligodendrogliomas, 1 grade II astrocytoma, 2 grade II oligoastrocytomas and 1 grade III oligoastrocytoma) (WHO classification 2007) and 9 BM (4 lung adenocarcinomas, 2 breast adenocarcinomas, 1 ovarian adenocarcinoma, 1 melanoma and 1 esophageal adenocarcinoma). Following histological examination, two cases demonstrated radionecrosis (case 23) or inflammatory tissue (case 26).

In each case, the quantity of resected tissue was evaluated as small (S) < 1cm^3^ or large (L) ≥ 1 cm^3^.

### Treatments

Thirteen patients who were diagnosed with glioma received radiotherapy (60 Gy) and chemotherapy before the [18F] FDOPA PET; one patient received chemotherapy only. All patients (n = 9) who had metastases were treated with radiotherapy (stereotactic radiotherapy 20 Gy) and all of these patients received chemotherapy before the [18F] FDOPA PET. Nine patients (5 gliomas and 4 metastases) were under chemotherapy at the time of [18F] FDOPA PET. All these data are detailed in S1 Table. The mean time from completion of radiation to the PET scan was 246.81 days (±159.18, range 58–744) and the mean time from the last cycle of chemotherapy to the PET scan was 203.21 days (± 447.35, range 0–1548). Fifteen patients experienced previous surgery of BT (biopsy or resection) at time of initial diagnosis and the mean interval between initial surgery and PET scan was 523.73 days (± 665.46, range 143–2413).

### [18F] FDOPA PET

The patients were given 100 mg of carbidopa orally one hour prior to the [18F] FDOPA administration. They were injected intravenously with 2 MBq/kg of [18F] FDOPA. PET/CT acquisition was started 20 minutes post injection and consisted in a 10 minutes static acquisition on a Biograph mCT (Siemens Healthcare, Erlangen, Germany). Images were reconstructed using an OSEM 5 iterations 24 subsets algorithm (with scatter and attenuation correction but without PSF compensation). For visual reading, [18F] FDOPA PET images were fused to contrast enhanced T1 weighted MR data acquired within 28 days [18F] FDOPA image analysis was performed using 3D volumes of interest (VOI). The VOI limits were determined using the Standard Uptake Value (SUV) contouring best the striatum. This value was used to define the contour level of the lesion’s VOI. A spherical background area was also used. We used different figures of merit (FOM) to express the [18F]-FDOPA uptake: SUVmax, SUVmean, SUVpeak and tumor uptake ratios (over striatum and background).

If a patient presented with several brain lesions (5 cases), only the uptake of the resected lesion was taken into account.

### Immunohistochemistry

LAT1 immunohistochemistry was performed on paraffin-embedded tumour sections using a rabbit monoclonal antibody for LAT1 (clone EPR3492, OriGen Technologies, 1:250) as previously described [17].

The subcellular localization of the staining was noted. For each case, a score of LAT1 expression based on the Hirsch score [19] was determined ranging between 0 and 400. The score was established on the whole tumour sample by a consensus between two observers. Microvessel staining was used both as a positive control and for the evaluation of staining intensity. Slides without primary antibody were used as negative control.

For each case, the density of vessels was quantified: the mean number of vessel per high power field (x 400) was calculated from the examination of 10 high power fields.

### Statistical analysis

Fisher’s exact test was used to compare qualitative data and the Wilcoxon signet-rank test for quantitative data. Before correlation analyses, a normality test (Shapiro-Wilk) showed that the variables were normal except for SUVpeak, SUVpeak T/S, SUVmax T/N, intervals CT-PET and intervals surgery-PET. Correlation between LAT1 score and the different SUV and correlation between intervals from treatment to PET scan and the different SUV were calculated using non parametric Spearman test. For comparisons between glioma grades, gliomas were divided into two groups: glioblastoma versus lower grade gliomas (grade II and III). Mann Whitney U has been used to compare the different SUV according to the grade and according to the presence or the absence of a chemotherapy during the PET scan. All the tests were considered significant at a 5% type I error rate (p<0.05). Statistical analyses were performed using SPSS version 11.0 (Statistical Package for Social Sciences, SPSS inc; Chicago, USA).

## Results

### Expression of LAT1 in brain tumors

**Fig 1:**
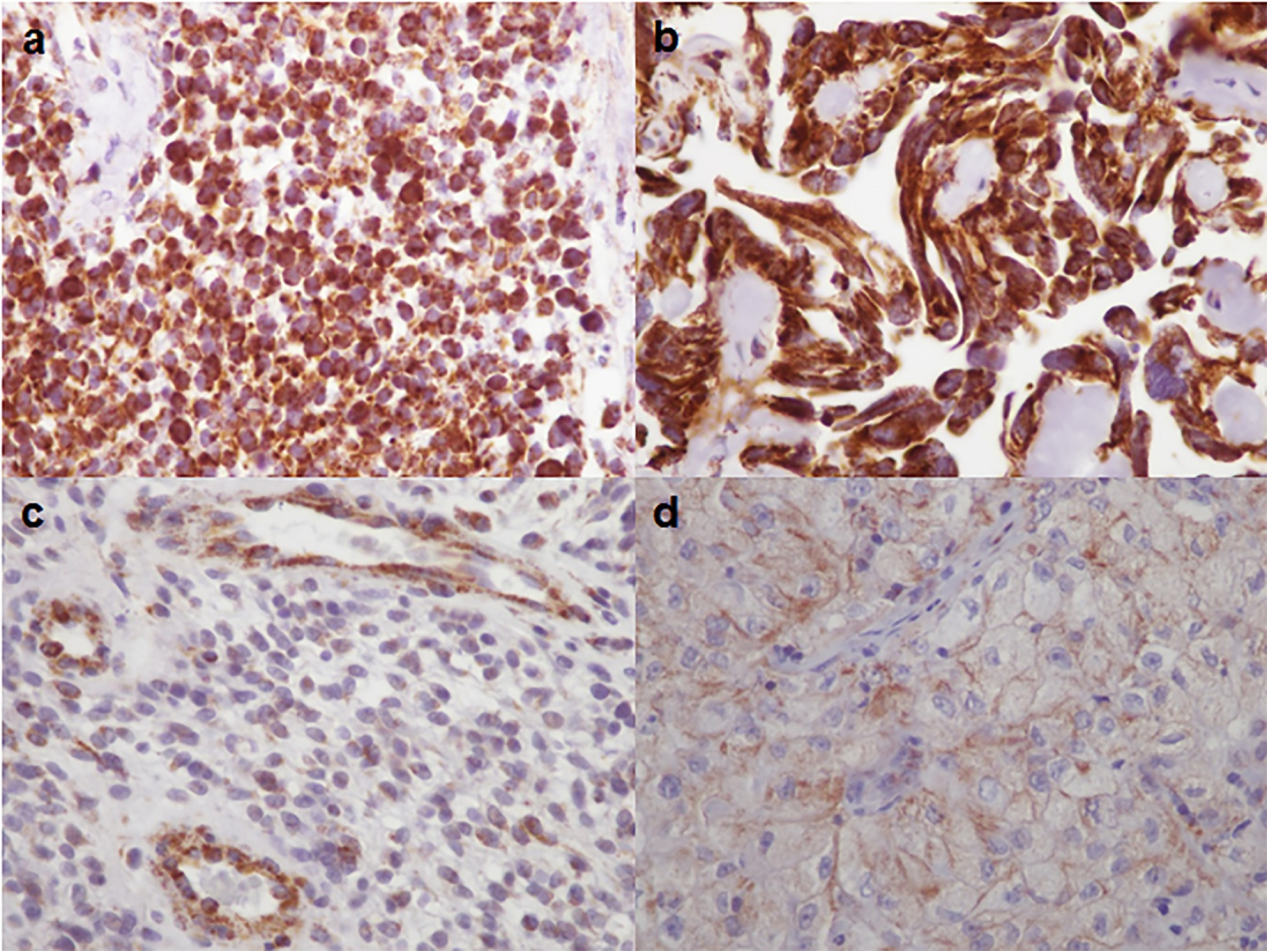
LAT1 immunolabelling (x400). Diffuse and strong LAT1 expression in a small cell glioblastoma (a) and in a metastasis of ovary adenocarcinoma (b). Weak LAT1 expression in a glioblastoma (c) and in a metastasis of lung adenocarcinoma (d). Note LAT1 expression in endothelial cells (c).

LAT1 expression was detected in all cases, with a score varying from 20 to 400 (mean score 185) (Fig 1). LAT1 staining was frequently detected both membranous and cytoplasmic patterns (20/28); rarely it was predominantly membranous (1 case) or in cytoplasm (7 cases) (Fig 1). Histological and immunohistological data are reported in Table 1. There was no difference between metastases and gliomas (p = 0.46).

**Table 1:** Histological characteristics and expression of LAT1 in brain tumours.

Case	Tumour type	Primitive site	Sample size	LAT1 score	Predominant subcellular localization	Vessels n/HPF	LAT1 staining intensity in vessels
1	Metastasis	breast	L	160	Cytoplasm	21	weak
2	Metastasis	lung	L	180	Membrane	6	moderate
3	Glioblastoma		L	190	Mixed	14	weak
4	Glioblastoma		L	100	Mixed	1	moderate
5	Oligo II		L	100	Mixed	9	weak
6	Glioblastoma		L	165	Cytoplasm	12	strong
7	Oligo II		L	240	Cytoplasm	6	weak
8	Glioblastoma		S	50	Mixed	11	moderate
9	Glioblastoma		L	180	Cytoplasm	9	weak
10	Metastasis	ovary	L	400	Mixed	16	weak
11	Glioblastoma		L	400	Mixed	28	weak
12	Metastasis	esophagus	L	400	Mixed	12	weak
13	Glioblastoma		L	150	Mixed	11	weak
14	Metastasis	lung	L	180	Cytoplasm	22	weak
15	Metastasis	lung	S	300	Cytoplasm	8	weak
16	Glioblastoma		L	285	Mixed	7	moderate
17	Metastasis	lung	L	270	Mixed	6	weak
18	Glioblastoma		S	300	Mixed	8	weak
19	Glioblastoma		S	160	Mixed	11	weak
20	Metastasis	breast	L	285	Mixed	17	weak
21	Oligoastro III		L	130	Mixed	13	moderate
22	Glioblastoma		S	40	Mixed	22	moderate
23	Metastasis (radionecrosis)	melanoma	L	20	Cytoplasm	18	weak
24	Oligoastro II		L	30	Mixed	5	weak
25	Oligoastro II		S	20	Mixed	4	weak
26	Astro II (inflammation)		L	90	Mixed	25	weak
27	Glioblastoma		L	160	Mixed	7	strong
28	Glioblastoma		L	200	Mixed	21	moderate

Oligo: oligodendroglioma, Oligoastro: Oligoastrocytoma, Astro: Astrocytoma. II: Grade II, III: Grade III. S: less than 1 cm^3^; L: more than 1 cm^3^. n/HPF: mean number of vessels per high power field (x400)

### Quantification of [18F] FDOPA uptake in brain tumours and correlation with LAT1 expression score

We searched as to whether LAT1 expression in BT correlated with intensity of [18F] FDOPA uptake. The different values of quantification are reported in S1 Table.

A significant [18F] FDOPA uptake defined by a ratio SUVmax T/S > 0.75 was present in 24 cases, 21 of these cases displayed a LAT1 score ≥ 100. Among the 4 cases showing a low [18F] FDOPA uptake (ratio SUVmax T/S ≤ 0.75), 3 cases displayed a LAT1 score <100. As previously observed [17], a significant [18F] FDOPA uptake was significantly associated with a LAT1 score ≥ 100 (p = 0.02), suggesting that a minimal expression of LAT1 is required for [18F] FDOPA uptake.

**Fig 2:**
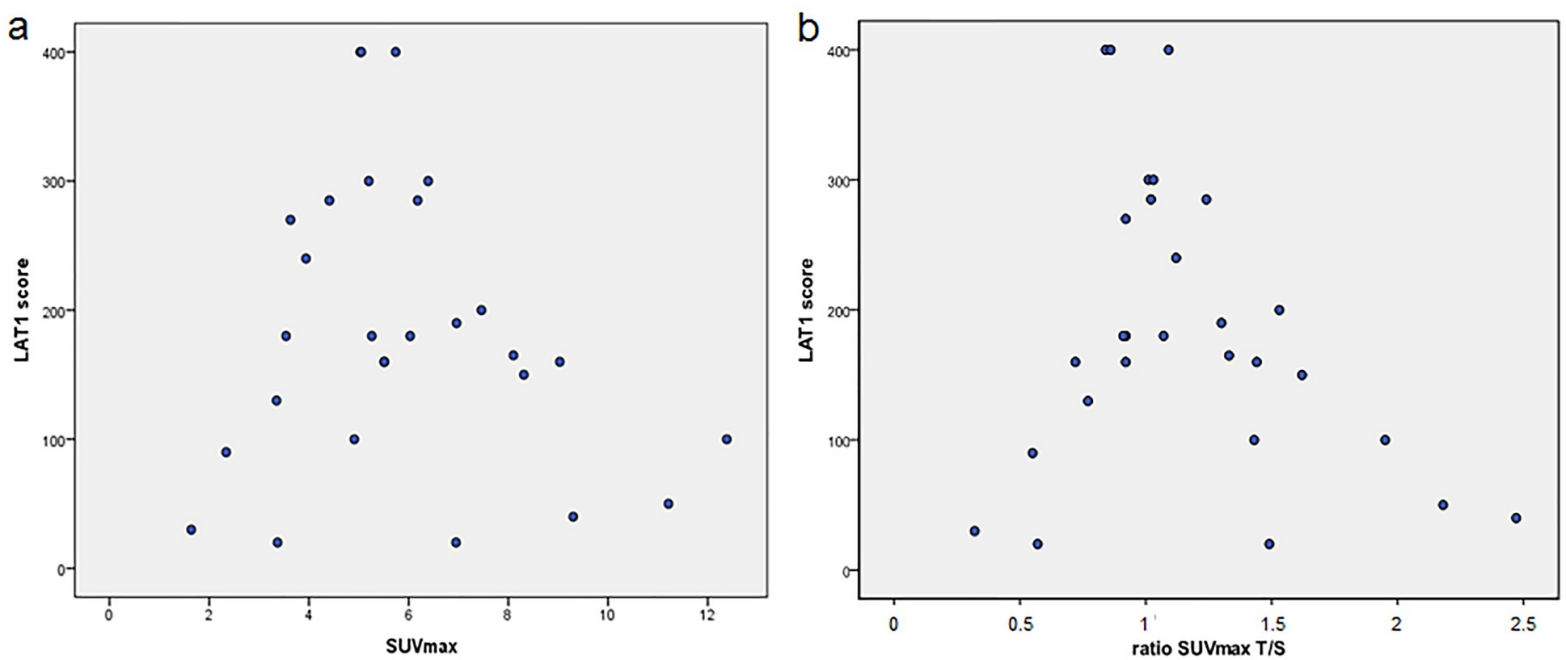
Correlation between LAT1 scores and [18F] FDOPA standardized uptake values. a) correlation between LAT1 scores and SUVmax of tumours. b) correlation between LAT1 scores and ratio SUVmax T/S.

Nevertheless, intensity of [18F] FDOPA uptake was not directly correlated to the score of LAT1 expression, whatever the value or the ratio considered (SUVmax, SUVmax T/S, SUVpeak T/S, SUVmax T/N and SUVmean T/S), with p = 0.7, p = 0.47, p = 0.28, p = 0.4 and p = 0.49 respectively (Fig 2). It suggests that the level of LAT1 expression in tumour cells alone is not sufficient to explain variations of [18F] FDOPA uptake among brain tumours.

There was no difference between the group of metastases and the group of gliomas. There was no significant difference between irradiated tumours (22 cases) and non irradiated tumors (6 cases).

**Fig 3:**
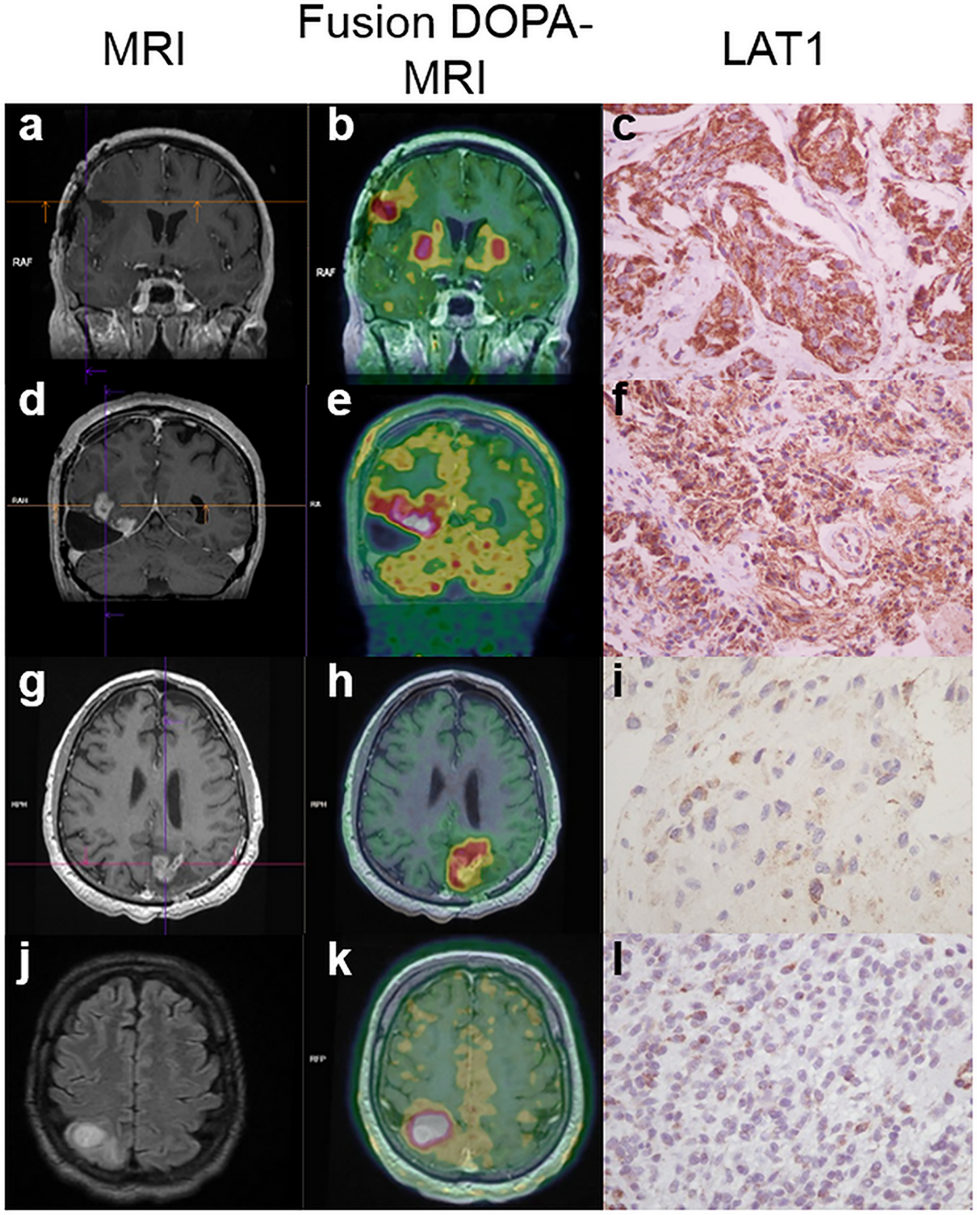
**Illustration of LAT1 score and [18F] FDOPA PET in four cases**: MRI (a,d,g,j), [18F] FDOPA-PET images fused with MRI (b, e, h, k) and LAT1 scores (c, f, i, l) of four cases are presented to illustrate the results. Case 20 (a, b, c) corresponding to a metastasis from breast carcinoma displayed a high SUVmax (6.18), a ratio SUVmax T/S of 1.24 and a high score of LAT1 expression (285). Case 28 (d, e, f) corresponding to a glioblastoma showed a high SUVmax (7.48), a ratio SUVmax T/S of 1.53 and a high LAT1 score (200). Case 8 (g, h, i) corresponding to a glioblastoma showed a high SUVmax (11.21), a ratio SUVmax T/S of 2.18 and a low LAT1 score (50). Case 22 (j, k, l) corresponding to a glioblastoma showed a high SUVmax (9.3), a ratio SUVmax T/S of 2.47 and a low LAT1 score (40).

It is noteworthy that in 8 cases we observed an important discordance between LAT1 expression and intensity of [18F] FDOPA uptake: some cases (10, 11, 12 and 18) showed a strong expression of LAT1 (score ≥300) and a weak [18F] FDOPA uptake (SUVmax <6). Conversely, some cases (4, 8, 22, 25) showed a weak LAT1 expression (score ≤ 50) and a strong [18F] FDOPA uptake (SUVmax >6) (Figs 2 and 3). The quantity of resected tissue was significantly smaller (<1 cm3, equivalent to a biopsy) in these highly discordant cases as compared to the other cases (p = 0.038) suggesting that the tissue sample may not be representative of the entire tumour. However, even if these cases were excluded from the analysis because of a lack of representativeness, no correlation between the score of LAT1 expression and the intensity of [18F] FDOPA uptake was found (p = 0.16).

These results suggest that the intensity of [18F] FDOPA uptake did not correlate with the level of LAT1 expression in tumours.

### Impact of histological parameters on intensity of [18F] FDOPA uptake

Intensity of [18F] FDOPA uptake was correlated with glioma grade: SUVmax was significantly higher in glioblastomas as compared to lower grade gliomas (mean SUVmax 7.15 versus 3.85, p = 0.008) as well as SUVmean values (3.36 versus 2.4, p = 0.035). LAT1 score was higher in glioblastomas than in lower grade gliomas but the difference was not statistically significant (mean LAT1 score 183 versus 101, p = 0.08).

Due to the fact that endothelial cells express LAT1, we also quantified the number of vessels in each case in order to determine whether intensity of [18F] FDOPA uptake may be influenced by vessels density.

Mean density of vessels per field (x400) varied from 1 to 28. Intensity of staining of LAT1 in vessels was moderate to strong in 9 cases and weak in 19 cases. The details are reported in Table 1. Both vessels density and staining intensity of LAT1 was not significantly different according to histological subgroup (metastasis vs glioma), with p = 0.15 and p = 1 respectively.

Intensity of [18F] FDOPA uptake did not correlate either with vessels density or with staining intensity of LAT1 in vessels (p = 0.7 and p = 0.21 respectively).

In conclusion, intensity of [18F] FDOPA uptake increased with glioma grade but was independent of vessel density.

### Impact of treatment on intensity of [18F] FDOPA uptake

In order to determine whether the intensity of [18F] FDOPA uptake may be influenced by treatment, we compared the different SUV according to the intervals from initial surgery, radiotherapy and chemotherapy. The ratio SUVmaxT/S and SUVmaxT/N were significantly related to the interval from chemotherapy (p = 0.03 and p = 0.02, respectively). We did not find significant correlation between the interval from surgery and from radiotherapy to PET scan (Table 2).

**Table 2:** Relation between intervals from treatment to PET scan and parameters of [18F] FDOPA uptake.

	Interval from radiotherapy		Interval from chemotherapy		Interval from surgery	
	r	p	r	p	r	p
SUVmax	-0.09	0.67	-0.22	0.29	-0.35	0.19
SUVpeak	-0.06	0.76	-0.18	0.4	-0.5	0.05
SUVmean	-0.12	0.59	-0.02	0.9	-0.39	0.14
SUVmaxT/S	-0.01	0.95	-0.43	**0.03**	+0.04	0.87
SUVmaxT/N	-0.15	0.48	-0.47	**0.02**	-0.1	0.71
SUVpeakT/S	+0.06	0.76	-0.33	0.12	+0.07	0.8
SUVmeanT/S	+0.22	0.32	-0.38	0.07	+0.05	0.84
LAT1 score	+0.19	0.39	-0.14	0.5	-0.44	0.09

Significant results are shown in bold.

SUV were not significantly modified by a chemotherapy during the PET scan (p>0.05).

These results showed a relationship between the interval from chemotherapy to the PET scan for SUVmaxT/S and SUVmaxT/N with a higher ratio being related to a smaller interval from chemotherapy.

## Discussion

The present study shows that a significant [18F] FDOPA uptake in brain tumours was significantly associated with a score of LAT1 expression higher than 100. This is in line with the generally accept premise that a minimal expression of LAT1 is required for significant [18F] FDOPA uptake. Nevertheless, intensity of [18F] FDOPA uptake did not appear to be linearly correlated with the level of LAT1 expression.

To the best of our knowledge this study is the largest series comparing intensity of [18F] FDOPA uptake and LAT1 expression in brain tumours. Only one previous study [15] has been performed and it reported a significant correlation between LAT1 expression and intensity of [18F] FDOPA uptake in a series of six gliomas prior to any treatment. In our study including 19 gliomas, we did not observe such a similar correlation. We used similar parameters to assess LAT1 expression and intensity of [18F] FDOPA uptake. Youland et al. quantified [18F] FDOPA uptake using SUVmean value, which was also evaluated in our study. SUVmean is highly dependent on the VOI delineation, therefore, we also tested other FOMs (SUVmax, SUVpeak and ratios). However, none of the FOM was correlated with level of LAT1 expression. In the study of Youland et al. stereotactic biopsies were [18F] FDOPA-PET guided allowing spatial correspondence between histological samples and [18F] FDOPA-PET images. In our study, the correlation between histological sample and [18F] FDOPA-PET image was not controlled. Most histological samples were large (>1cm^3^) but six samples were small and were potentially subject to sampling errors. However the exclusion of small samples in which the sampling biases could be the highest did not change the results. The tumours types in the two studies differed with regard to their treatment. In the study of Youland *et al*., none of the patients diagnosed with a glioma received treatment prior to PET whereas in our study most tumours represented recurrences and had been previously irradiated. The effects of irradiation on LAT1 expression in tumours and on [18F] FDOPA uptake are unknown. Chiaravalloti *et al*. [20] showed that [18F] FDOPA SUV may be affected by treatment, and in particular, by radiotherapy. They demonstrated a significant relationship between the interval from RT to the PET/CT scan for both SUVmax and SUVmean with a higher SUV being related to a smaller interval from RT. In our study, we did not find such a significant correlation with the interval from RT to the PET scan, possibly due to the small size of our series, but we find a correlation with the intervals from CT, strengthening the idea that treatments may impact on PET outcome. It is possible that irradiation or chemotherapy may induce changes in cell metabolism, especially in AA metabolism. Irradiation classically induces important histological changes such as necrosis and inflammation. Cases of inflammatory lesion (one case of neurosarcoidosis and one case of acute disseminated encephalomyelitis) showing [18F] FDOPA uptake in PET have been reported, suggesting that inflammation may also play a role in [18F] FDOPA uptake [21]. Mechanisms which are responsible for the [18F] FDOPA uptake in inflammatory conditions are unknown and remain to be elucidated. While LAT1 has been shown to be the main transporter of [18F] FDOPA in tumour cells, there is no data concerning the transport of [18F] FDOPA in inflammatory cells. Such studies have been performed for other radiopharmaceuticals AA or analogs, in particular [11C]-MET and [18F] FACBC. Oka S *et al*. [7] showed that inflammatory cells were able to uptake [18F] FACBC and that the transport system (Na+ dependent versus Na+ independent) varied according to cell type (tumour cells versus inflammatory cells) and to the pH of the medium. *In vitro*, [18F] FACBC uptake by inflammatory cells was mediated by Na+ dependent system and was potentiated in alkaline medium while tumour cells used Na+ independent (one of whose main representatives is LAT1) which is potentiated in acid medium. With regard to [18F] FDOPA, it has not been excluded that transporters other than LAT1 may be involved in [18F] FDOPA uptake by inflammatory cells. Such a hypothesis might explain why there is no direct correlation between level of LAT1 expression and intensity of uptake in [18F] FDOPA–PET, particularly in irradiated tumors which are likely to harbor necrotic and inflammatory changes.

In our previous study on brain metastases [17] and in the present study, a significant [18F] FDOPA uptake (defined by a ratio SUVmax T/S >0.75) in tumours was correlated with a LAT1 score higher than 100, suggesting that, as expected, LAT1 expression may play an important role in [18F] FDOPA uptake and that a minimal LAT1 expression is required for [18F] FDOPA uptake. The lack of direct correlation between the level of LAT1 expression and the level of intensity of [18F] FDOPA uptake in our study may be due to several reasons: sampling and intra-tumour heterogeneity, small size of our series and heterogeneity of tumours (gliomas and metastases), interferences with other factors influencing [18F] FDOPA uptake such as tumour grade [22–24] and treatments [20].

In conclusion, although our study showed that a positive [18F] FDOPA uptake in PET was associated with a minimal threshold of LAT1 expression, we did not find a linear correlation between intensity of [18F] FDOPA uptake and level of LAT1 expression. These results remain compatible with a role of LAT1 in [18F] FDOPA uptake by brain tumours but suggest that LAT1 expression alone is not sufficient to explain variation of intensity of [18F] FDOPA uptake in BT. These should be confirmed in a larger and more homogeneous series of tumours in the light of other potential factors influencing [18F] FDOPA uptake.

## Supporting information

S1 Table Characteristics of tumors, treatments, LAT1 scores and standardized uptake values. SUVmax T/S: ratio SUVmax tumor/ SUVmax contralateral striatum, SUVmax T/N: ratio SUVmax tumor/ SUVmax background; SUVpeak T/S: ratio SUVpeak tumor/ SUVpeak contralateral striatum; SUVmean T/S: SUVmean tumor / SUVmean contralateral striatum.
(XLSX)
